# FADS2 confers SCD1 inhibition resistance to cancer cells by modulating the ER stress response

**DOI:** 10.1038/s41598-024-64043-2

**Published:** 2024-06-07

**Authors:** Toshikatsu Ikeda, Yuki Katoh, Hirotsugu Hino, Daichi Seta, Tadashi Ogawa, Takashi Iwata, Hiroshi Nishio, Masaki Sugawara, Shuichi Hirai

**Affiliations:** 1 Division of Anatomical Science, Department of Functional Morphology, Nihon University School of Medicine, 30-1 Ohyaguchi-Kami-Cho, Itabashi-ku, Tokyo, 173-8610 Japan; 2 Department of Obstetrics and Gynecology, Keio University School of Medicine, 35 Shinano-machi, Shinjuku-ku, Tokyo, 160-8582 Japan; 3 Department of Legal Medicine, Aichi Medical University School of Medicine, 1-1 Yazakokarimata, Nagakute, Aichi 480-1195 Japan

**Keywords:** Stearoyl-CoA desaturase 1 (SCD1), Fatty acid desaturase 2 (FADS2), Endoplasmic reticulum (ER) stress, Palmitic acid, Biochemistry, Cancer, Molecular biology, Oncology

## Abstract

Stearoyl-CoA desaturase 1 (SCD1) is an attractive target for cancer therapy. However, the clinical efficacy of SCD1 inhibitor monotherapy is limited. There is thus a need to elucidate the mechanisms of resistance to SCD1 inhibition and develop new therapeutic strategies for combination therapy. In this study, we investigated the molecular mechanisms by which cancer cells acquire resistance to endoplasmic reticulum (ER) stress-dependent cancer cell death induced by SCD1 inhibition. SCD1 inhibitor-sensitive and -resistant cancer cells were treated with SCD1 inhibitors in vitro, and SCD1 inhibitor-sensitive cancer cells accumulated palmitic acid and underwent ER stress response-induced cell death. Conversely, SCD1-resistant cancer cells did not undergo ER stress response-induced cell death because fatty acid desaturase 2 (FADS2) eliminated the accumulation of palmitic acid. Furthermore, genetic depletion using siRNA showed that FADS2 is a key determinant of sensitivity/resistance of cancer cells to SCD1 inhibitor. A549 cells, an SCD1 inhibitor-resistant cancer cell line, underwent ER stress-dependent cancer cell death upon dual inhibition of SCD1 and FADS2. Thus, combination therapy with SCD1 inhibition and FADS2 inhibition is potentially a new cancer therapeutic strategy targeting fatty acid metabolism.

## Introduction

Stearoyl-CoA desaturase 1 (SCD1) is an endoplasmic reticulum (ER)-resident enzyme and a critical regulator of the fatty acid metabolic pathway. SCD1 is involved in the synthesis of monounsaturated fatty acids (MUFAs) such as palmitoleic acid (16:1 n-7) and oleic acid (18:1 n-9) from saturated fatty acids such as palmitic acid (16:0) and stearic acid (18:0)^1–3^. SCD1 is highly expressed in several types of cancers^4^ and has long been an attractive target in cancer therapy because it promotes cancer cell proliferation, tumor growth, and metastasis by maintaining cell membrane components^3,5,6^, modulating signal pathways such as the Wnt/β-catenin axis^7,8^, and regulating ER stress responses^9–12^. However, clinical trial results have shown that the efficacy of SCD1 inhibitors is limited and depends on the type of cancer and the patient^13^. There is thus a critical need to obtain a better understanding of the mechanism of SCD1 inhibitor resistance in order to develop new therapeutic strategies.

Fatty acid desaturase 2 (FADS2), a fatty acid desaturation enzyme, catalyzes the first rate-limiting step in the biosynthesis of polyunsaturated fatty acids (≥ 20 carbon fatty acids with ≥ 3 double bonds)^14^. Recent reports have shown that FADS2 performs a specialized metabolic function in cancer cells, synthesizing sapienic acid from palmitic acid^15^. It was also reported that this metabolic process is a mechanism by which cancer cells resist death caused by the depletion of plasma membrane components resulting from SCD1 inhibition. Specifically, SCD1 inhibitor-resistant cancer cells were shown to compensate for the depletion of MUFA, a component of the plasma membrane, via sapienic acid produced by FADS2, and its elongation product, cis-8-octadecenoate^15^. However, cancer cell death by SCD1 inhibition is regulated by multiple signals, such as an enhanced ER stress response from palmitate accumulation^16,17^, and the mechanism of resistance to SCD1 inhibitors remains to be fully elucidated.

Here, we investigated the mechanism by which cancer cells acquire tolerance to SCD1 inhibition, focusing on the association between fatty acid metabolic plasticity by FADS2 and the ER stress pathway.

## Results

### SCD1 inhibitor-resistant cancer cells exhibit increased metabolism of palmitic acid to sapienic acid because of the expression of FADS2

**Figure 1:**
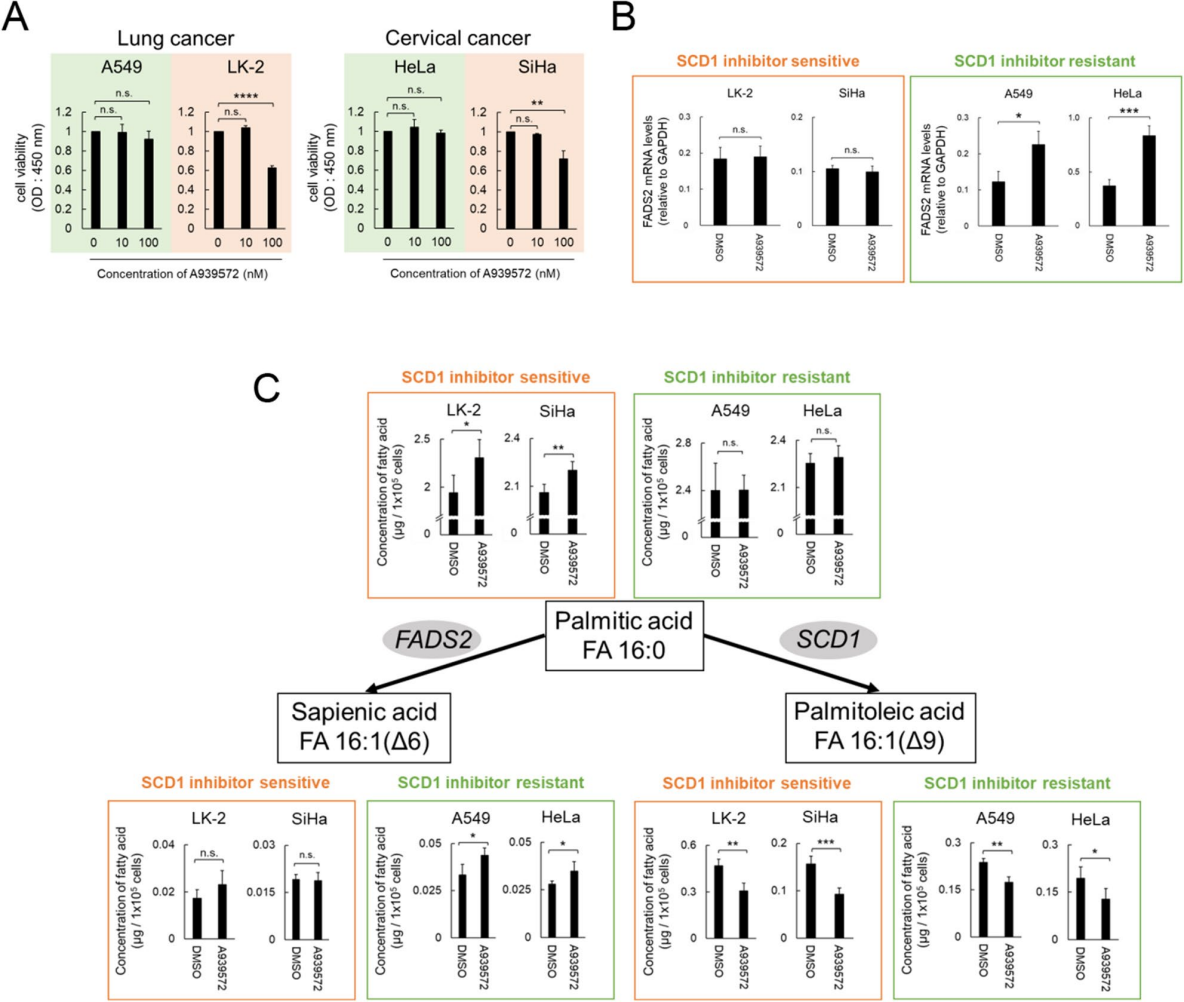
SCD1 inhibitor-resistant cancer cells desaturate palmitic acid to sapienic acid via FADS2. (**A**) Cancer cell lines were treated with the SCD1 inhibitor A939572 (0, 10, 100 nM). Cell viability was assessed by WST-1 assay. (**B**,**C**) Cancer cells were cultured in RPMI medium or DMEM containing 0.5% serum supplemented with 100 nM A939572 or dimethylsulfoxide (DMSO). (**B**) Total RNA was extracted and FADS2 mRNA was evaluated using qRT-PCR. (**C**) Palmitic, palmitoleic, and sapienic acid levels were evaluated using GC–MS. Data are expressed as means ± SD (n = 3). *P < 0.05, **P < 0.01, ***P < 0.001. *n.s*. not significant.

While SCD1 is a promising target in cancer therapy, the efficacy of SCD1 inhibitors varies by cancer type and patient^13^. To elucidate the mechanisms behind the differences in sensitivity to SCD1 inhibitor in cancer cells, we treated various cancer cell lines (lung cancer: A549 and LK-2; cervical cancer: HeLa and SiHa), in which SCD1 is expressed (Fig. S1A), with the SCD inhibitor A939572 or SCD1-specific siRNA under conditions of low extracellular fatty acid availability. Proliferation assays showed that the cells exhibited different sensitivity profiles to SCD1 inhibition (Fig. 1A and Fig. S2A,B). LK-2 and SiHa cells were classified as “SCD1 inhibitor-sensitive,” while A549 and HeLa cells were classified as “SCD1 inhibitor-resistant.”

Next, we explored the mechanisms by which cancer cells differ in their sensitivity to SCD1 inhibitors. SCD has two isoforms in humans, SCD1 and SCD5^2^, and because SCD5 may complement the function of SCD1, we first tested the involvement of SCD5 in sensitivity to SCD1 inhibitors. Expression analysis of SCD5 in all four cell lines showed that its expression was very low at baseline compared with that of SCD1 and was not increased by SCD1 inhibitor treatment. In addition, there was no correlation between sensitivity to SCD1 inhibitor and SCD5 expression (Fig. S1B). These findings suggested that SCD1 inhibitor sensitivity/resistance is not determined by SCD5.

Interestingly, previous reports have shown that the production of sapienic acid following FADS2 expression results in loss of sensitivity to SCD1 inhibitors^15^. Therefore, we evaluated the expression of FADS2 in SCD1 inhibitor-sensitive or -resistant cancer cells treated with SCD1 inhibitor or SCD1-specific siRNA. Consistent with the previous report^15^, the SCD1 inhibitor-resistant cancer cell lines, A549 and HeLa, showed increased FADS2 in response to SCD1 inhibition (Fig. 1B and Fig. S2C).

We subsequently assessed fatty acid levels in SCD1 inhibitor-sensitive/resistant cancer cells. Inhibition of SCD1 decreased the amount of palmitoleic acid, a SCD1-generated fatty acid, in the four cancer cell lines (Fig. 1C and Fig. S2D). Notably, LK-2 and SiHa cells accumulated palmitic acid and A549 and HeLa cells accumulated sapienic acid (Fig. 1C and Fig. S2D). These results strongly suggest that cancer cells that can metabolize palmitic acid to sapienic acid by FADS2 when treated with SCD1 inhibitors may acquire resistance to SCD1 inhibitor.

### SCD1 inhibitor sensitivity is dependent on the ER stress state of cancer cells

**Figure 2:**
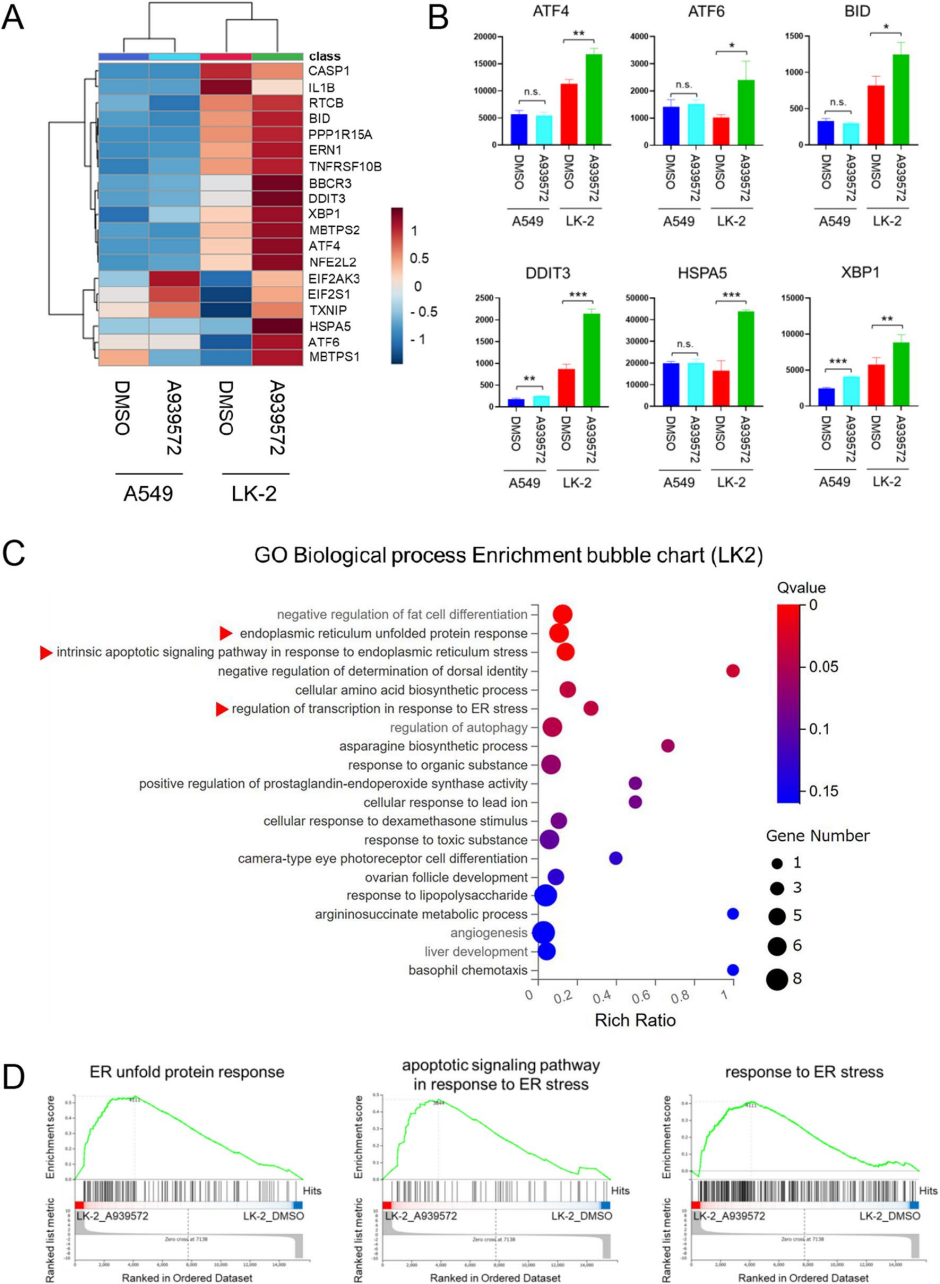
ER stress state upon SCD1 inhibition differs between SCD1 inhibitor-sensitive and -resistant cancer cells. SCD1 inhibitor-sensitive cancer cells (LK-2) and SCD1 inhibitor-resistant cancer cells (A549) were cultured in RPMI medium containing 0.5% serum supplemented with A939572 or DMSO. Total RNA was extracted from cells and RNA-seq was performed. (**A**) A heat map of ER stress-related genes. (**B**) Comparison of expression levels of genes of interest [ER stress markers (ATF4, ATF6, BID, DDIT3, HSPA5, and XBP1)]. (**C**) The top 20 GO terms of the transcripts upregulated by SCD1 inhibitor treatment in LK-2 cells are shown. Red arrowheads indicate ER stress-related pathways. (**D**) GSEA was used to evaluate the changes in ER stress-related pathways in SCD1 inhibitor-treated LK-2 cells. Data are expressed as means ± SD (n = 3). *P < 0.05, **P < 0.01, ***P < 0.001. *n.s.* not significant.

SCD1 inhibitors induce the accumulation of saturated fatty acids such as palmitic acid and stearic acid and promote the death of cancer cells by enhancing ER stress^16,17^. To investigate the association between different fatty acid metabolic profiles and ER stress status, we performed RNA-seq analysis of LK-2 cells, an SCD1 inhibitor-sensitive cancer cell line, and A549 cells, an SCD1 inhibitor-resistant cancer cell line. The expression of ER stress-related genes such as ATF4, ATF6, BID, DDIT3, HSPA5, and XBP1 was upregulated in LK-2 cells by SCD1 inhibitor treatment, while A549 cells had a lower degree of upregulation (Fig. 2A,B). The RNA-seq analysis results were confirmed by RT-qPCR (Fig. S3A). Gene Ontology (GO) analysis and Gene Set Enrichment Analysis (GSEA) were also performed to validate that a functional ER stress response occurred upon SCD1 inhibitor treatment, which confirmed enhancement of the ER stress-related pathway in LK-2 cells, but not in A549 cells (Fig. 2C,D and Fig. S4). In addition, gene expression of spliced XBP1 (sXBP1) and protein expression of GRP78, phosphorylated eIF2α and CHOP, functional markers of the ER stress response, were also markedly elevated in LK-2 cells while showing smaller change in A549 cells (Fig. S3B,C). Thus, we speculate that SCD1 inhibitor-resistant cancer cells may utilize FADS2-mediated metabolism of palmitic acid to avoid cell death caused by the ER stress response.

### FADS2 confers resistance to SCD1 inhibitor-induced ER stress-dependent cell death in cancer cells

**Figure 3:**
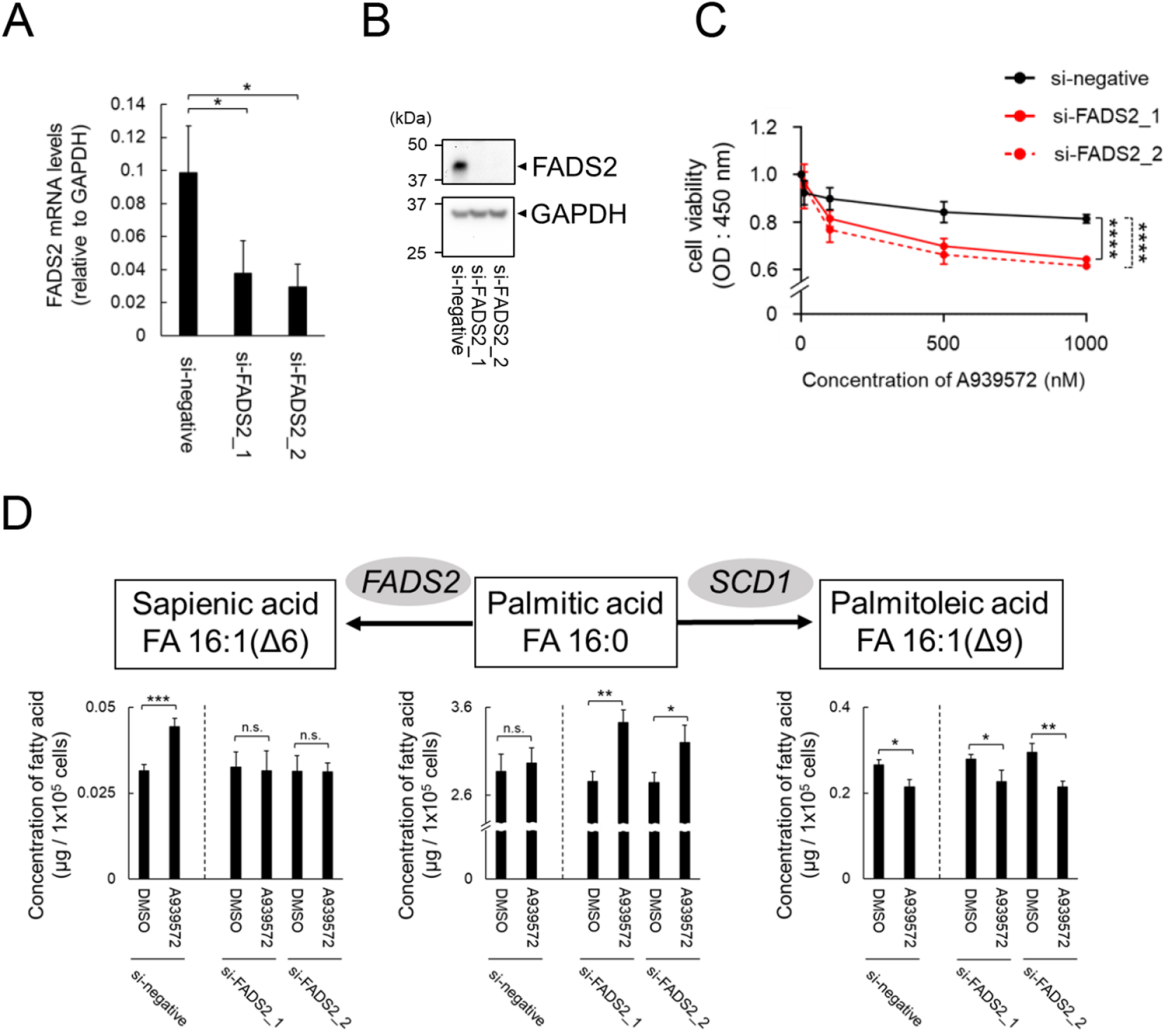
Knockdown of FADS2 reverses SCD1 inhibitor-induced cell death and fatty acid metabolic profiles. SCD1 inhibitor-resistant A549 cells were transfected with small interfering RNA (siRNA)-FADS2 or siRNA-control (si-negative). (**A**) FADS2 mRNA levels were evaluated by qRT-PCR at 48 h post-transfection. (**B**) FADS2 protein levels were evaluated by western blotting. Original blots are presented in Figure S6. (**C**) A549 cells transfected with siRNA were treated with A939572 (0, 10, 100, 500, 1000 nM). Cell viability was assessed by WST-1 assay. (**D**) The levels of palmitic acid, palmitoleic acid, and sapienic acid in A549 cells transfected with siRNA were evaluated using GC–MS. Data are expressed as means ± SD (n = 3). *P < 0.05, **P < 0.01, ***P < 0.001, ****P < 0.0001. *n.s.* not significant.

**Figure 4:**
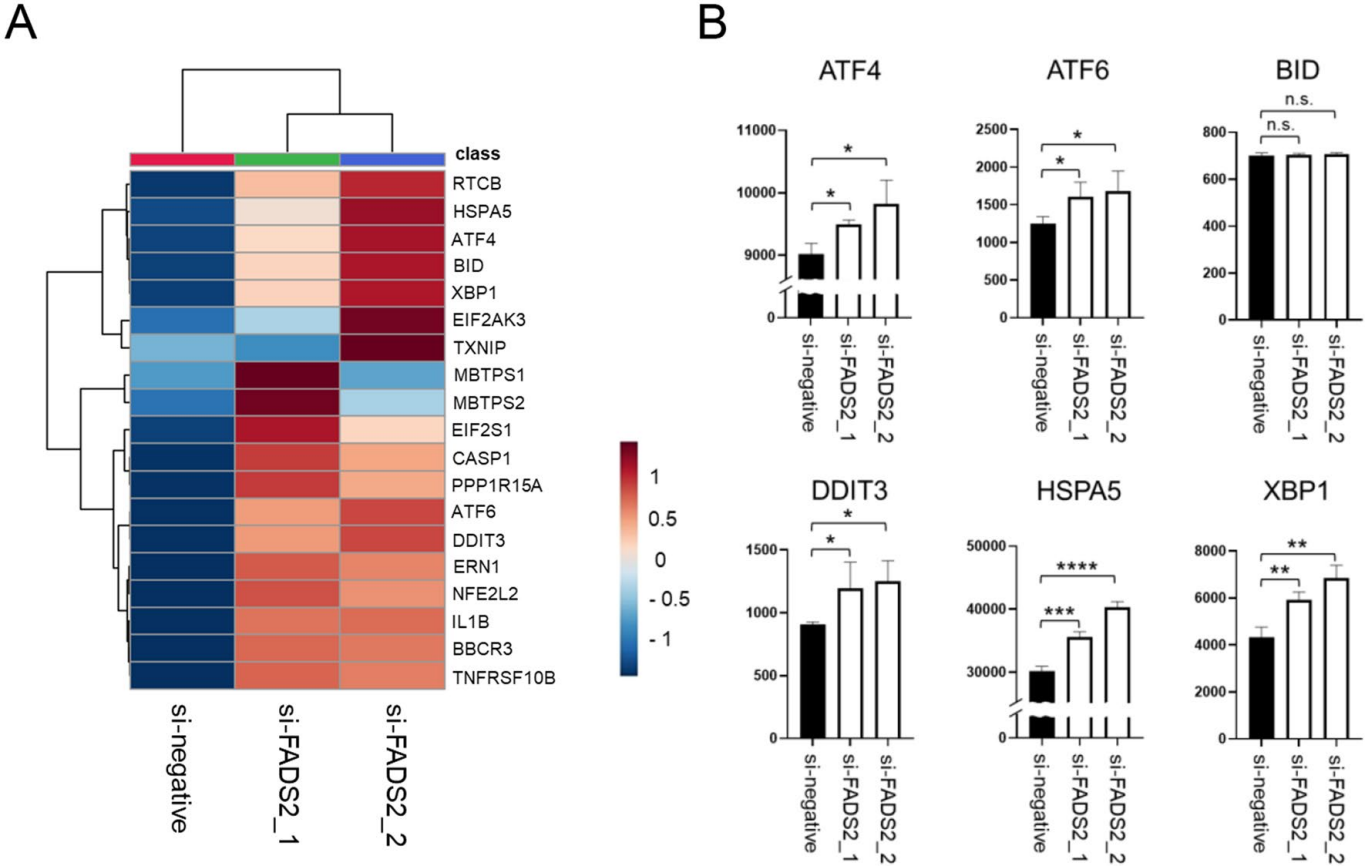
Knockdown of FADS2 enhances the ER stress response induced by SCD1 inhibitor. Total RNA was extracted from A549 cells transfected with siRNA-FADS2 or siRNA-control and treated with SCD1 inhibitor, and RNA-seq was performed. (**A**) A heat map of ER stress-related genes. (**B**) Comparison of expression levels of genes of interest [ER stress markers (ATF4, ATF6, BID, DDIT3, HSPA5, and XBP1)].

To investigate the association of FADS2 with the mechanism of ER stress-mediated SCD1 inhibitor sensitivity/resistance, we performed FADS2 knockdown by siRNA in A549 cells, an SCD1-resistant cancer cell line (Fig. 3A,B). A549 cells with FADS2 knockdown showed altered cellular characteristics in response to SCD1 inhibitors, including suppressed cell proliferation and loss of sapienic acid accumulation (Fig. 3C,D). Because the accumulation of palmitic acid was observed in FADS2-knockdown A549 cells, RNA-seq analysis was performed to assess ER stress status. The results showed that the ER stress response was enhanced by SCD1 inhibitor treatment in FADS2-knockdown A549 cells (Fig. 4A,B). These results suggest that quantitative changes in intracellular fatty acids by FADS2 may regulate cell death induced by ER stress caused by SCD1 inhibition and partially determine sensitivity to SCD1 inhibitors.

## Discussion

**Figure 5:**
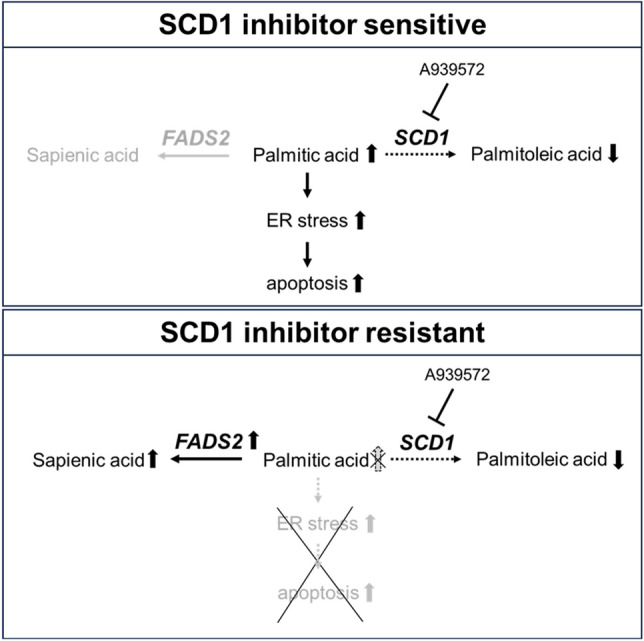
Schematic of the proposed mechanism. SCD1 inhibitor-resistant cancer cells escape ER stress-dependent cell death by avoiding palmitic acid accumulation via FADS2.

Recently, many drugs targeting cancer metabolism have entered the clinical trial phase^13^. SCD1, a key molecule in fatty acid metabolism, has been recognized as a promising target for anticancer drugs because of accumulating evidence that it is important for tumorigenesis. However, while SCD1 inhibitors have shown antitumor effects in vitro and in in vivo mouse models, the results in clinical trials have been poor. Therefore, to improve the efficacy of SCD1 inhibitors as anticancer agents, it is necessary to elucidate the mechanisms of resistance to them and to identify concomitant drugs that enhance therapeutic efficacy. In this study, we revealed a mechanism of SCD1 inhibitor resistance in which an alternative metabolic pathway by FADS2 cancels the ER stress-dependent cell death caused by SCD1 inhibition due to the accumulation of palmitic acid. Furthermore, dual inhibition of SCD1 and FADS2 may be an effective therapeutic strategy for SCD1 inhibitor-resistant cancer cells (Fig. 5).

To date, various SCD1 inhibitors (e.g. A939572, CAY10566, MF-438, and CVT-11127) have successfully reduced cancer cell viability in vitro and tumor growth in vivo for several cancer types, including renal, prostate, breast, lung, endometrial, and colon cancer^18–24^. However, the results of clinical trials of SCD1 inhibitors have been poor, suggesting the existence of a mechanism of resistance to these inhibitors. Vriens et al. previously reported an SCD1 inhibitor resistance mechanism in which SCD1 inhibitor-resistant cancer cells complement the depletion of MUFAs by FADS2-mediated generation of sapienic acid and its elongation product, cis-8-octadecenoate^15^. In this study, we demonstrated that SCD1 inhibitor-sensitive and -resistant cancer cells differ in their ER stress status. Furthermore, FADS2-knockdown experiments showed that FADS2 is an important factor that controls the ER stress state of cancer cells when SCD1 is inhibited. Thus, as FADS2 is involved in the escape of multiple mechanisms of cancer cell death induced by SCD1 inhibition, the expression of FADS2 in tumor tissue may be a biomarker for patients undergoing SCD1 inhibitor therapy.

Previous studies reported that high concentrations of palmitic acid induce apoptosis in various types of cancer cells, including human liver cancer cells, HER2-positive breast cancer cells, and colon cancer cells, via induction of the ER stress response^25–27^. A recent report showed that the enhancement of ER stress by palmitic acid not only induces apoptosis, but also releases calcium from the endoplasmic reticulum, increases cytosolic calcium levels, and regulates transferrin transport, thereby affecting intracellular iron content and inducing ferroptosis-like cell death^26^. In this study, we demonstrated that SCD1 inhibitor-sensitive cancer cells exhibited increased palmitic acid levels in response to SCD1 inhibition, resulting in ER stress-induced apoptosis; in contrast, SCD1 inhibitor-resistant cancer cells showed no accumulation of palmitic acid and ER stress was not induced. Additionally, SCD1 inhibitor-resistant cancer cells showed increased sapienic acid levels because of the increased expression of FADS2. These results suggest that some cancer cells acquired the capacity to resist SCD1 inhibitor by escaping ER stress-induced apoptosis through the accumulation of palmitic acid via the upregulation of FADS2 expression. Furthermore, measurements of SCD1-related fatty acids, oleic acid and stearic acid, showed that SCD1 inhibitor treatment decreased oleic acid levels and did not change stearic acid levels in both SCD1 inhibitor-sensitive and -resistant cancer cells (Fig. S5A,B). Stearic acid may be metabolized by ELOVL1/3 to arachidic acid and lignoceric acid, so its accumulation and consequent induction of ER stress response may not occur. Thus, it was suggested that ER stress-dependent cell death is induced by the accumulation of palmitic acid, but not stearic acid, supporting our conclusion. Taking these findings together, therapeutic strategies that aid in increasing intracellular palmitate concentrations, such as FADS2 inhibition, may be important to maximize the clinical efficacy of SCD1 inhibitors as anticancer agents.

The limited number of cancer cell lines and SCD1 inhibitors used was a limitation of this study. A939572 is one of the most commonly used SCD1 inhibitors with high specificity, but needs more extensive validation. Nonetheless, the implications of our study are noteworthy.

In summary, FADS2 may be a key factor in determining SCD1 inhibitor sensitivity/resistance in cancer cells via regulation of the palmitic acid-induced ER stress response. Therapeutic interventions targeting FADS2 and SCD1/FADS2-related fatty acids may be an attractive therapeutic strategy to potentiate the efficacy of SCD1 inhibitors as anticancer agents.

## Methods

### Cells, cell culture, and reagents

The HeLa and SiHa human cervical cancer cell lines and A549 and LK-2 human lung cancer cell lines were purchased from the American Type Culture Collection and maintained in our laboratory. HeLa and SiHa cells were cultured in Dulbecco’s modified Eagle’s medium (DMEM) containing 10% heat-inactivated fetal bovine serum (FBS), 100 U/ml penicillin, and 100 µg/ml streptomycin. A549 and LK-2 cells were cultured in RPMI 1640 containing 10% FBS, 100 U/ml penicillin, and 100 µg/ml streptomycin. The concentration of FBS in the SCD1 inhibition experiments was 0.5% or 2%. Cell lines were authenticated by short tandem repeat profile analysis (Promega, Madison, WI, USA). All cancer cell lines were verified to be negative for mycoplasma contamination using the PCR Mycoplasma Detection Set (TaKaRa, Shiga, Japan) prior to experiments. A939572, an SCD1 inhibitor, was purchased from APExBIO (Houston, TX, USA).

### RNA extraction and RT-qPCR analysis

Total RNA was isolated from cultured cells using an RNeasy Mini Kit (Qiagen, Hilden, Germany). TaqMan RT-PCR primers and probes for human SCD1, FADS2, ATF4 and DDIT3 were purchased from Integrated DNA Technologies. SYBR Green RT-PCR primers for human sXBP1 and SCD5 were purchased from Sigma Aldrich Sigma Aldrich/Merck KGaA, (Darmstadt, Germany) and FASMAC (Kanagawa, Japan), respectively (Table S1). Relative gene expression was determined using the 2^−ΔCt^ method, where ΔCt is the difference between the mean Ct value of triplicate measurements of the sample and the endogenous GAPDH control.

### Western blotting

Samples were extracted using radio-immunoprecipitation assay (RIPA) lysis buffer containing 50 mM Tris–HCl (pH 8.0), 150 mM NaCl, 1.0% Nonidet P-40, 0.5% sodium deoxycholate, 0.1% SDS, protease inhibitor cocktail (Nacalai Tesque, Kyoto, Japan), and phosphatase inhibitor cocktail (EDTA free) (Nacalai Tesque). Protein concentrations were measured using the Protein Assay Bicinchoninate kit (Nacalai Tesque). Equal amounts of proteins were loaded on a gel, separated by SDS–polyacrylamide gel electrophoresis, and transferred onto Immobilon-P membranes (Millipore/Merck KGaA, Darmstadt, Germany). The membranes were blocked with 3% skim milk (Nacalai Tesque) and probed with the following primary antibodies: mouse or rabbit anti-FADS2 antibody (68026-1-Ig; Proteintech, Rosemont, IL, USA), mouse anti-SCD1 antibody (ab19862; Abcam, Cambridge, UK), rabbit anti-BiP/GRP78 antibody (#3177; Cell Signaling Technology, Danvers, MA, USA), rabbit anti-phosphorylated eIF2α (Ser51) antibody (#3398; Cell Signaling Technology), rabbit anti-eIF2α antibody (#9722; Cell Signaling Technology), mouse anti-CHOP antibody (#2895; Cell Signaling Technology), and mouse anti-GAPDH antibody (sc-32233; Santa Cruz Biotechnology, Dallas, TX, USA) at room temperature for 1 h. After washing three times with TBST (25 mM Tris–HCl, 137 mM NaCl, 2.7 mM KCl, 0.05% Tween20, pH7.4), the blots were incubated with goat anti-mouse immunoglobulins/HRP (Dako/Agilent Technologies, Santa Clara, CA, USA) or swine anti-rabbit immunoglobulins/HRP (Dako/Agilent Technologies) secondary antibodies at room temperature for 1 h. Signals were detected using Immobilon Forte Western HRP (Millipore/Merck KGaA) on a Molecular Imager ChemiDoc XRS (Bio-Rad Laboratories, Tokyo, Japan). Signal intensity of each band was quantified using CS Analyzer 4 software (ATTO Corporation, Tokyo, Japan).

### WST-1 assay

Cancer cell viability was evaluated using WST-1 Solution Reagent (Roche Diagnostics, Mannheim, Germany) following the manufacturer’s instructions.

### Sample preparation for gas chromatography-mass spectrometry (GC–MS)

Cells were sonicated in 0.3 mL of PBS containing internal standards (100 ng of margaric acid). Fatty acids were extracted using an ISOLUTE SLE + column and dichloromethane. The organic fractions were dried under a nitrogen stream. The residue was dissolved in 5 µL of pyridine and 30 µL of BSTFA + TMCS (99:1) (TS-38831; Thermo Fisher Scientific, Waltham, MA, USA) for trimethylsilylation. The derivatization reaction was performed for 30 min at 40 °C^7^.

### GC–MS analysis

GC–MS analysis was performed on a Shimadzu GC–MS QP2010 Ultra equipped with an AOC20i autoinjector and Rtx-5MS column (30 m, 0.25 mm, 0.25 µm df) in 70 eV electron ionization mode. The temperature program was as follows: 150 °C for 1 min, 20 °C/min to 250 °C, 5 °C/min to 280 °C, hold for 5 min, 20 °C/min to 330 °C, and hold for 3 min when the temperature was maintained for 10 min. The carrier gas was helium with a constant flow rate of 42.0 cm/s. One microliter was injected in a 5:1 split ratio at an injector temperature of 250 °C. The MS interface temperature was held at 280 °C. Selected ion monitoring for quantification was performed by recording the ions at m/z 311.2 for sapienic acid-trimethylsilyl derivative, m/z 311.2 for palmitoleic acid-trimethylsilyl derivative, m/z 313.2 for palmitic acid-trimethylsilyl derivative, m/z 327.2 for margaric acid-trimethylsilyl derivative, m/z 339.2 for oleic acid-trimethylsilyl derivative, and m/z 341.2 for stearic acid-trimethylsilyl derivative^28^. The four evaluated fatty acids are summarized in Table 1.

**Table 1:** The gas chromatography-mass spectrometry (GC/MS) analyses of free fatty acids.

Compound	Target ion, *m/z*	Rt^(1)^ (min)	LOD^(2)^ (ng/20μL)	LOQ^(3)^ (ng/20μL)
Sapienic acid	311.2	5.15	0.11	0.26
Palmitoleic acid	311.2	5.75	0.13	0.32
Palmitic acid	313.2	5.83	0.09	0.22
Oleic acid	339.2	6.67	0.09	0.24
Stearic acid	341.2	6.79	0.08	0.21
Margaric acid	327.2	6.29	I.S	I.S

^(1)^Retention time, ^(2)^Limit of detection, ^(3)^Limit of quantification.

### RNA sequencing

RNA sequencing was performed using a commercial service (BGI, Huada Gene, Wuhan, China). In brief, total RNA was fragmented into short fragments, mRNA was enriched using oligo(dT) magnetic beads, and cDNA synthesis was performed. Double-stranded cDNA was purified and enriched by PCR amplification, and library products were sequenced using BGIseq-500. Kyoto Encyclopedia of Genes and Genomes (KEGG) pathway and GO bioinformatic analyses were performed using the Dr. Tom data mining system (BGI, Huada Gene).

### Gene knockdown

FADS2-specific (50 nM/L; Sigma Aldrich), SCD1-specific (100 nM/L; Thermo Fisher Scientific) or control small interfering RNAs (siRNAs) (50 nM/L; Sigma Aldrich) were transfected into cancer cells using Lipofectamine. The primer sequences are shown in Supplementary Table S2. Cells were collected after incubation for 72 h.

### Statistical analysis

Statistical analyses were performed using GraphPad Prism 9 software. Comparisons between two groups were made using unpaired or paired (for matched comparisons) two-tailed Student’s t-tests or the non-parametric Mann–Whitney *U*-test. Multiple comparisons were made by one-way ANOVA with Tukey’s multiple comparisons test. Data are presented as means ± SD. P < 0.05 was considered statistically significant.

### Supplementary Information

Supplementary Information.

## Data Availability

The datasets used and/or analyzed during the current study are available from the corresponding author on reasonable request.

## Abbreviations

SCD1: Stearoyl-CoA desaturase 1
ER: Endoplasmic reticulum
FADS2: Fatty acid desaturase 2
MUFA: Monounsaturated fatty acid
GSEA: Gene set enrichment analysis
DMEM: Dulbecco’s modified Eagle’s medium
FBS: Fetal bovine serum
STR: Short tandem repeat
GC–MS: Gas chromatography-mass spectrometry
KEGG: Kyoto encyclopedia of genes and genomes
siRNA: Small interfering RNA
