# Supplementary material for: FADS2 confers SCD1 inhibition resistance to cancer cells by modulating the ER stress response
Source: Sci Rep. 2024 Jun 7;14:13116. doi: 10.1038/s41598-024-64043-2 (PMC11161504; doi:10.1038/s41598-024-64043-2)
Supplement: Supplementary file 1 — Supplementary Information. [file 41598_2024_64043_MOESM1_ESM.pdf]

Title: FADS2 confers SCD1 inhibition resistance to cancer cells by modulating the ER stress response.

Toshikatsu Ikeda[1#], Yuki Katoh[1,2#*], Hirotsugu Hino[1], Daichi Seta[1], Tadashi Ogawa[3], Takashi Iwata[2], Hiroshi Nishio[2], Masaki Sugawara[2], Shuichi Hirai[1]

Supplemental Figure 1-7

Supplemental Table 1 and 2

Supplemental Figure 1

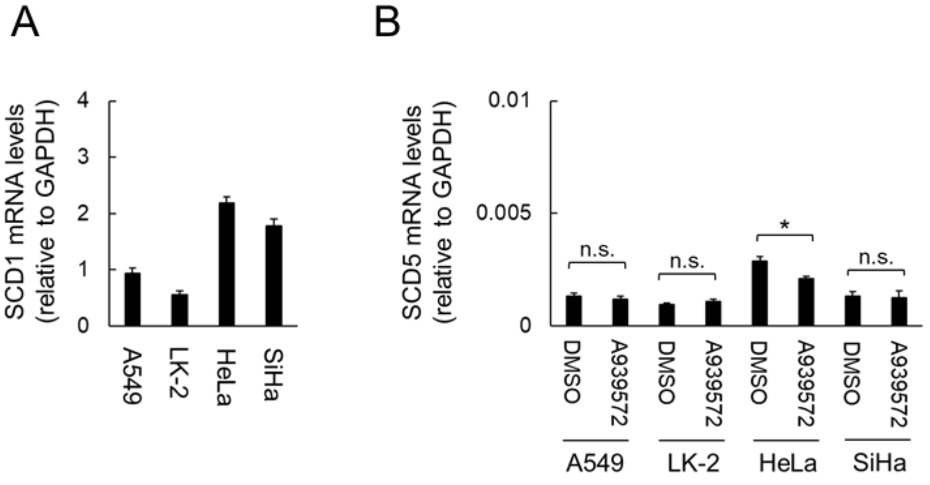

Supplemental Figure 1. Gene expression of SCD1 and SCD5 in human lung cancer cell lines and cervical cancer cell lines. (A) SCD1 mRNA expression was analyzed by qRT-PCR in A549, LK-2, HeLa, and SiHa cells. **(B)** SCD5 mRNA expression before and after SCD1 inhibitor treatment in four cell lines.

Supplemental Figure 2

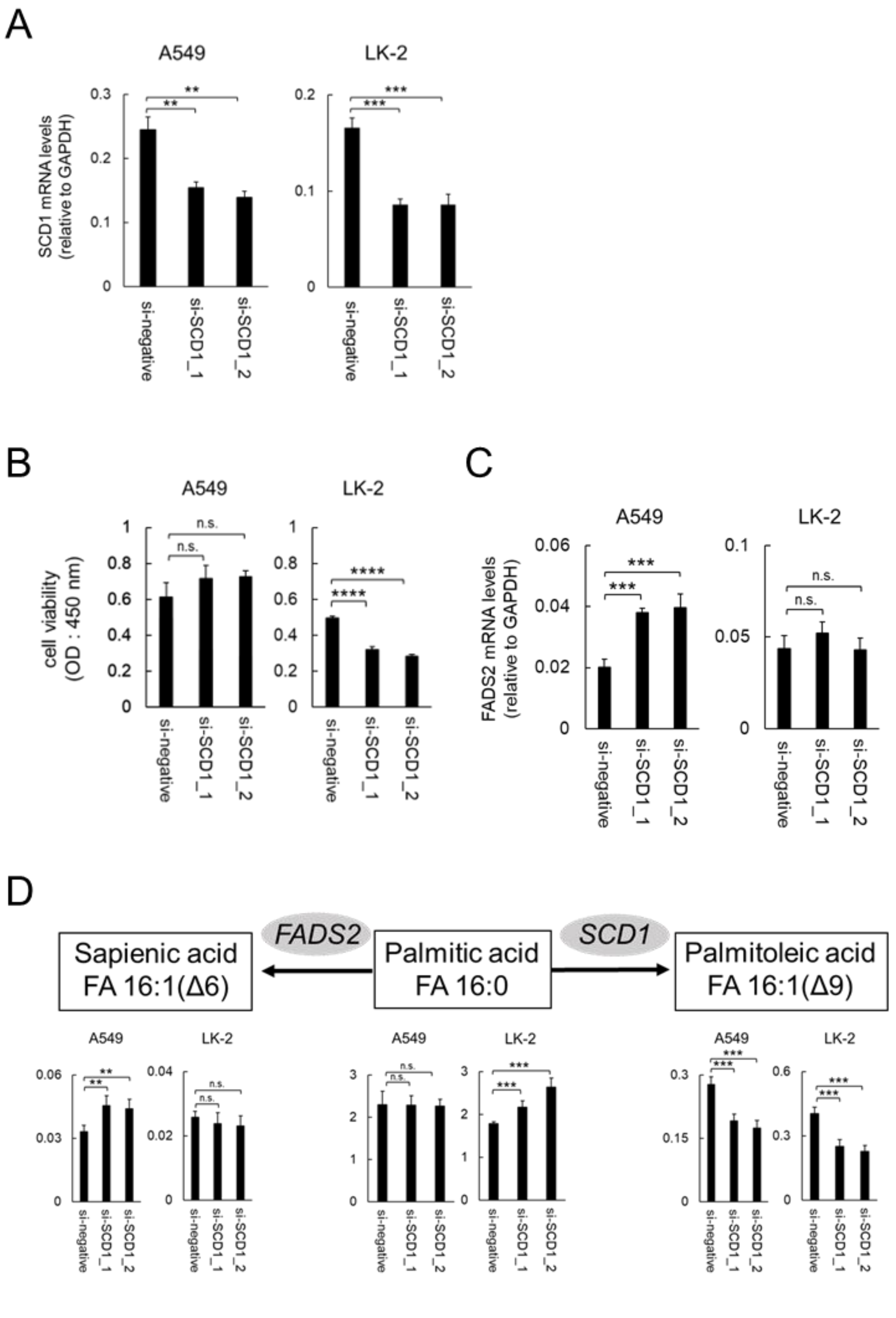

Supplemental Figure 2. Cancer cells resistant to SCD1 knockdown desaturate palmitate to sapienic acid via FADS2. A549 and LK-2 cells were transfected with small interfering RNA (siRNA)-

SCD1 or siRNA-control (si-negative). **(A)** SCD1 mRNA levels were evaluated by qRT-PCR at 48 h post-transfection. **(B)** Cell viability was assessed by WST-1 assay. **(C)** FADS2 mRNA levels were evaluated using qRT-PCR. **(D)** Palmitic, palmitoleic, and sapienic acid levels were evaluated using GC-MS. Data are expressed as means ± SD (n=3). $^{**}P<0.01$, $^{***}P<0.001$, $^{****}P<0.0001$. n.s., not significant.

Supplemental Figure 3

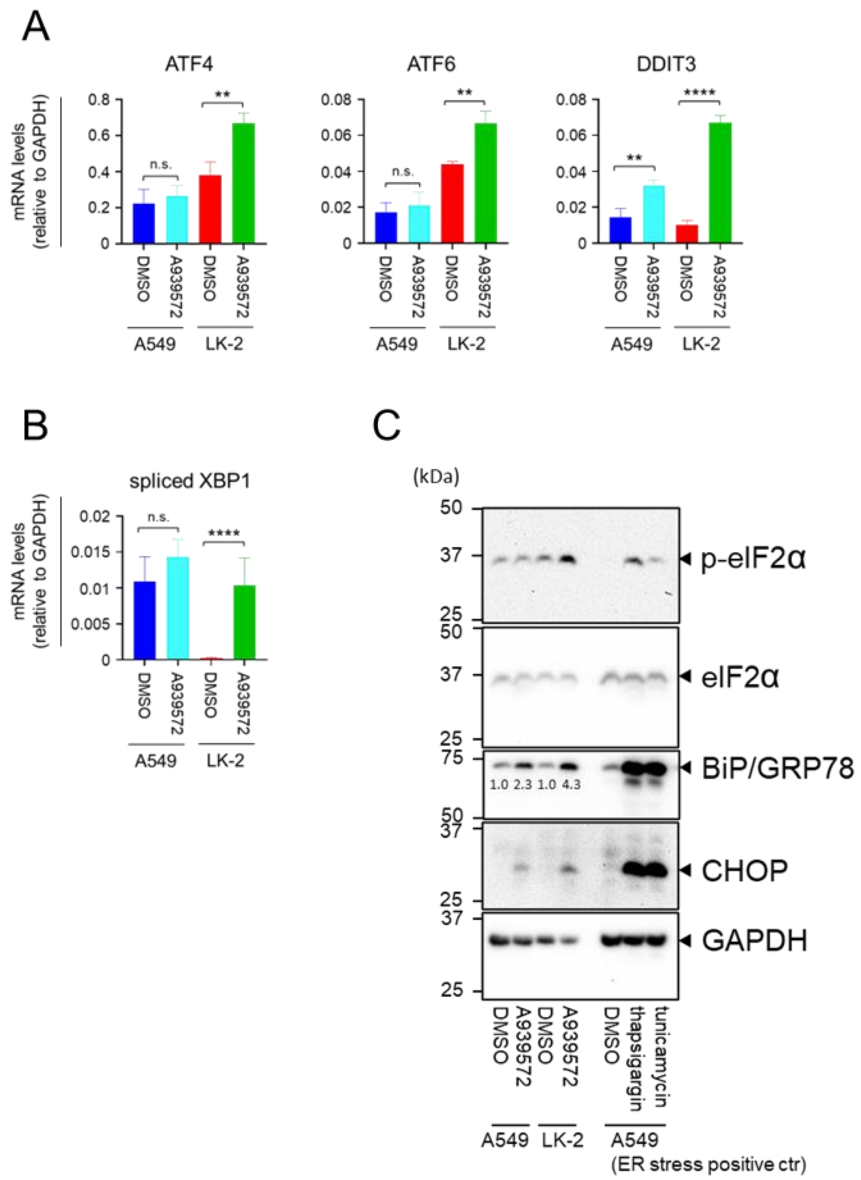

Supplemental Figure 3. SCD1 inhibitor-induced ER stress response differs between SCD1 inhibitor-sensitive and -resistant cancer cells. A549 and LK-2 cells were cultured in RPMI medium containing 0.5% serum supplemented with A939572 or DMSO. **(A, B)** Total RNA was extracted and

RT-qPCR was performed. Comparison of gene expression of ATF4, ATF6, and DDIT3 related to Figure 2A, B **(A)** and spliced XBP1, a functional ER stress response marker **(B)**. **(C)** Comparison of protein expression of GRP78, phosphorylated eIF2α and CHOP, functional markers of the ER stress response. Numbers in the figure indicate relative protein expression (GRP78/GAPDH). Data are expressed as means ± SD (n=3). **P<0.01, ****P<0.0001. n.s., not significant.

Supplemental Figure 4

GO Biological process Enrichment bubble chart (A549)

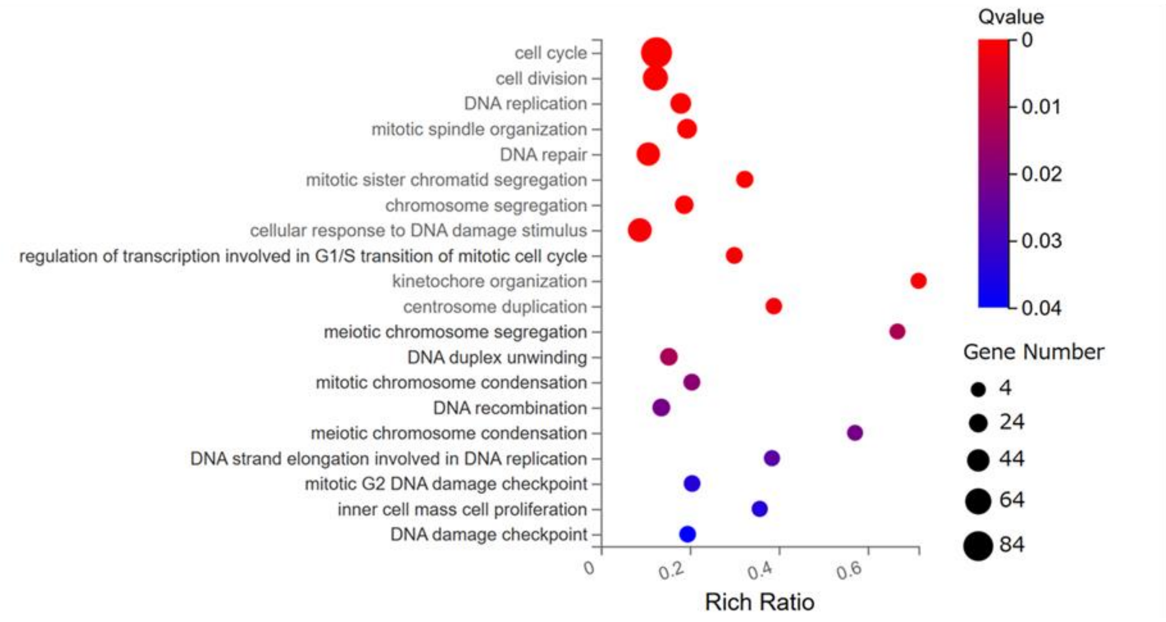

Supplemental Figure 4. SCD1 inhibitor-resistant cancer cells do not have an enhanced ER stress response to SCD1 inhibitor treatment. SCD1 inhibitor-resistant cancer cells (A549) were cultured in RPMI medium containing 2% serum supplemented with A939572 or DMSO. Total RNA was extracted and RNA-seq was performed. The top 20 GO terms of the transcripts upregulated by SCD1 inhibitor treatment in A549 cells are shown.

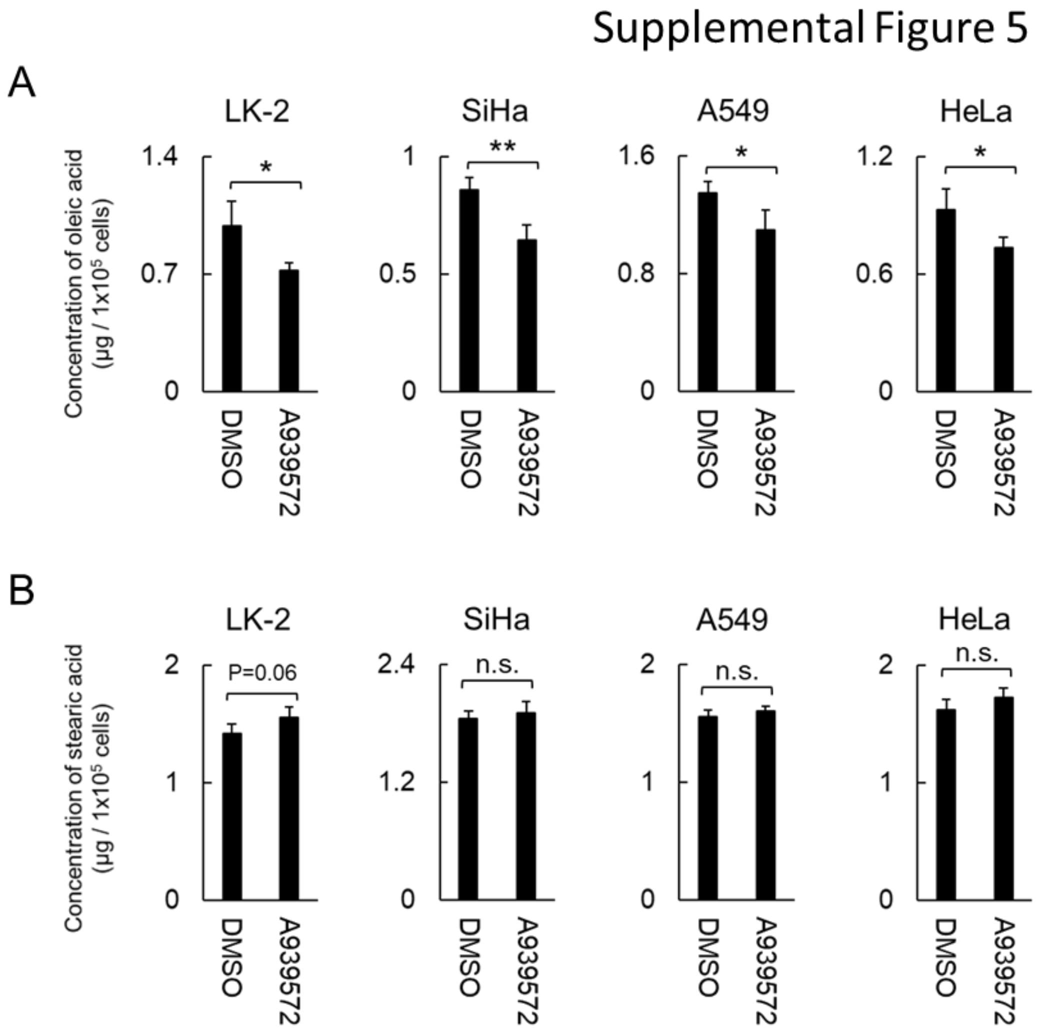

Supplemental Figure 5. No correlation between SCD1 inhibitor sensitivity and oleic and stearic acid levels in cancer cells. Cancer cells were cultured in RPMI medium or DMEM containing 0.5% serum supplemented with 100 nM A939572 or dimethylsulfoxide (DMSO). Oleic acid **(A)** and stearic acid **(B)** levels were evaluated using GC-MS. Data are expressed as means ± SD (n=3). *P<0.05, **P<0.01. n.s., not significant.

Supplemental Figure 6

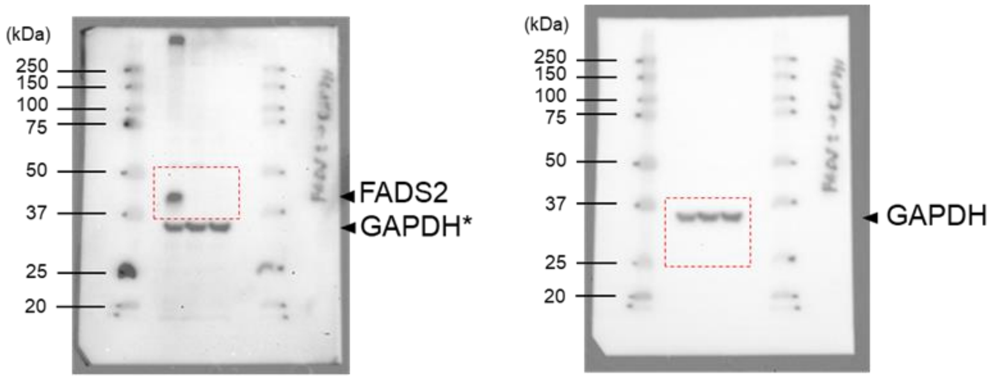

*: residual signal after stripping

Supplemental Figure 6. Western blot results uncropped and merged with corresponding marker images (Figure 3B). Merging of chemiluminescent signals and corresponding marker images was performed using Bio-Rad software Image Lab 6.0.

Supplemental Figure 7

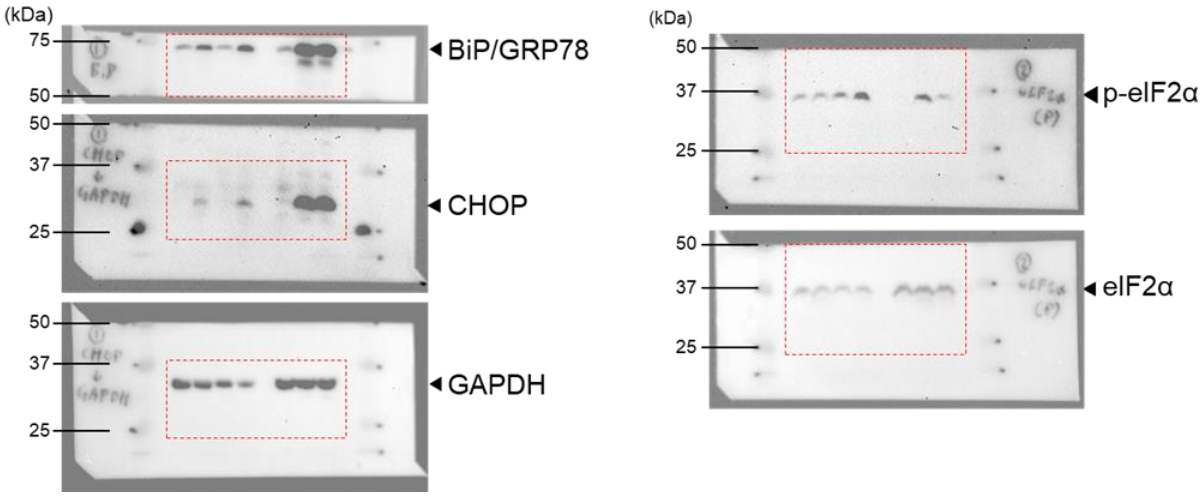

Supplemental Figure 7. Western blot results uncropped and merged with corresponding marker images (Figure S3C). Merging of chemiluminescent signals and corresponding marker images was performed using Bio-Rad software Image Lab 6.0.

sXBP1	forward	GCTGAGTCCGCAGCAGGT
	reverse	CTGGGTCCAACTTGTCCAGAAT
SCD5	forward	GAGGAATGTCGTCCTGATGAGC
	reverse	GCCAGGAGGAAGCAGAAGTAGG

Supplemental table 1. List of primers for human target gene.

si-negative	Sense	siRNA Universal Negative Control (sigma)
	Antisense	siRNA Universal Negative Control (sigma)
si-FADS2_1	Sense	CCAUGAUCGUCCAUAAGAA
	Antisense	UUCUUAUGGACGAUCAUGG
si-FADS2_2	Sense	GACAUGAACCUGUUCAAGA
	Antisense	UCUUGAACAGGUUCAUGUC
si-SCD1_1	Sense	CCAGAGGAGGUACUACAAAtt
	Antisense	UUUGUAGUACCUCCUCUGGaa
si-SCD1_2	Sense	CCCUGUAUGGGAUCACUUUtt
	Antisense	AAAGUGAUCCCAUACAGGGct

Supplemental table 2. List of si-RNAs for human target gene.
